# Combination of transcriptomic and proteomic approaches helps to unravel the protein composition of *Chelidonium majus* L. milky sap

**DOI:** 10.1007/s00425-016-2566-7

**Published:** 2016-07-11

**Authors:** Robert Nawrot, Jakub Barylski, Rico Lippmann, Lothar Altschmied, Hans-Peter Mock

**Affiliations:** 1 Department of Molecular Virology, Institute of Experimental Biology, Faculty of Biology, Adam Mickiewicz University in Poznań, Umultowska 89, 61-614 Poznań, Poland; 2 Leibniz Institute of Plant Genetics and Crop Plant Research (IPK), Corrensstrasse 3, 06466 Gatersleben, Germany; 3 Sandoz GmbH, Biochemiestraße 10, 6250 Kundl, Austria

**Keywords:** Greater celandine, Latex, Laticifers, Major latex protein, Papaveraceae, Polyphenol oxidase, Sequencing

## Abstract

**A novel annotated** ***Chelidonium majus*** **L. transcriptome database composed of 23,004 unique coding sequences allowed to significantly improve the sensitivity of proteomic** ***C. majus*** **assessments, which showed novel defense-related proteins characteristic to its latex.**

To date, the composition of *Chelidonium majus* L. milky sap and biosynthesis of its components are poorly characterized. We, therefore, performed *de novo* sequencing and assembly of *C. majus* transcriptome using Illumina technology. Approximately, 119 Mb of raw sequence data was obtained. Assembly resulted in 107,088 contigs, with N50 of 1913 bp and N90 of 450 bp. Among 34,965 unique coding sequences (CDS), 23,004 obtained CDS database served as a basis for further proteomic analyses. The database was then used for the identification of proteins from *C. majus* milky sap, and whole plant extracts analyzed using liquid chromatography–electrospray ionization-tandem mass spectrometry (LC–ESI-MS/MS) approach. Of about 334 different putative proteins were identified in *C. majus* milky sap and 1155 in *C. majus* whole plant extract. The quantitative comparative analysis confirmed that *C. majus* latex contains proteins connected with response to stress conditions and generation of precursor metabolites and energy. Notable proteins characteristic to latex include major latex protein (MLP, presumably belonging to Bet v1-like superfamily), polyphenol oxidase (PPO, which could be responsible for browning of the sap after exposure to air), and enzymes responsible for anthocyanidin, phenylpropanoid, and alkaloid biosynthesis.

**Electronic supplementary material:**

The online version of this article (doi:10.1007/s00425-016-2566-7) contains supplementary material, which is available to authorized users.

## Introduction

**Fig. 1:**
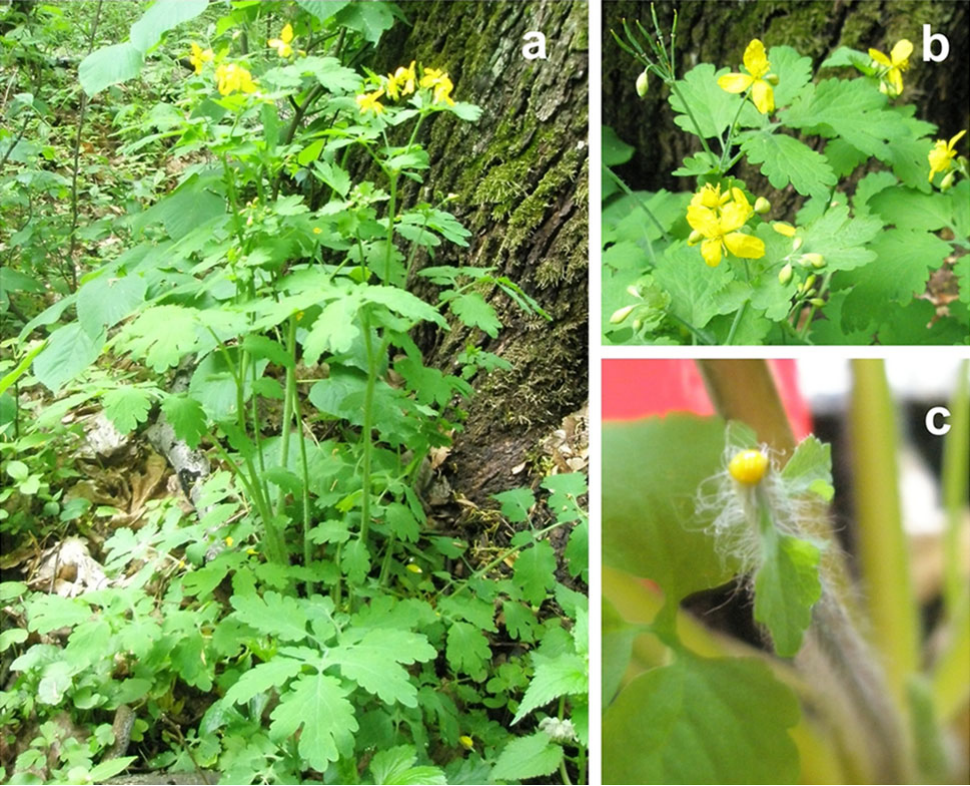
*Chelidonium majus* L. plant. **a** Adult plant grown in natural habitat during flowering. **b** *Yellow* flowers. **c** 6-week-old stem of the plant with exuding yellow milky sap, which was cut and used for RNA isolation

Greater celandine (*Chelidonium majus* L.) (Fig. 1), an herbaceous perennial plant, belongs to Papaveraceae family, which is an important source of biologically active substances. Milky sap and extracts of greater celandine are used in traditional medicine to treat papillae, warts, and condylomas, which are symptoms of human papilloma virus (HPV) infections. *C. majus* extracts are also used to treat liver disorders and fight fever (Hiller et al. 1998). The medicinal and pharmaceutical interest in this plant is based on its ability to synthesize alkaloids, flavonoids, or phenolic acids (Colombo and Bosisio 1996). It is often suspected that many of these substances may be either synthesized or stored in laticifers of the plant. These specialized cells produce milky sap (latex) that exudes when the plant is injured (Hagel et al. 2008). They are located in phloem areas forming an internal, articulated, and non-anastomosing system throughout the whole plant (Hagel et al. 2008). Therefore, sequestration of bioactive compounds into laticifers might protect the plant from the effects of its own toxins and provide a defense against herbivores (Hagel et al. 2008; Konno 2011; Escalante-Perez et al. 2012).

Previous proteomic studies regarding *C. majus* milky sap using two-dimensional electrophoresis (2-DE) and liquid chromatography–electrospray ionization-tandem mass spectrometry (LC–ESI-MS/MS) revealed the presence of about 21 proteins, mostly of defense- and pathogenesis-related properties (Nawrot et al. 2007a). However, sensitivity of used methods suffered from lack of relevant database of reference sequences.

*Chelidonium majus* (Fig. 1) belongs to non-model plants, and to date, its genome is unsequenced. Since assembly of such genome could be very laborious and correct gene annotation would require either homology-based or transcriptomic information, we decided to use RNA sequencing to create such database. Recent advances in next-generation sequencing (NGS) with the possibility of quantification of transcript abundances have greatly lowered its cost and provided insight into diverse plant species, including those with medicinal properties (Hao et al. 2011; Hamilton and Buell 2012). The *de novo* transcriptome sequencing and characterization has been performed successfully for *Taxus wallichiana* var. *mairei* (Lemée & H.Lév.) L.K.Fu & Nan Li (Hao et al. 2011), *Phalaenopsis* spp. (orchid) (Fu et al. 2011), *Macleaya* spp. (plume poppies) (Zeng et al. 2013), *Beta vulgaris* L. (sugarbeet) (Fugate et al. 2014), *Solanum tuberosum* L. (potato) (Gong et al. 2015), *Panax ginseng* C.A.Mey. (Jayakodi et al. 2015), *Salvia miltiorrhiza* Bunge (Xu et al. 2015), and many others.

**Fig. 2:**
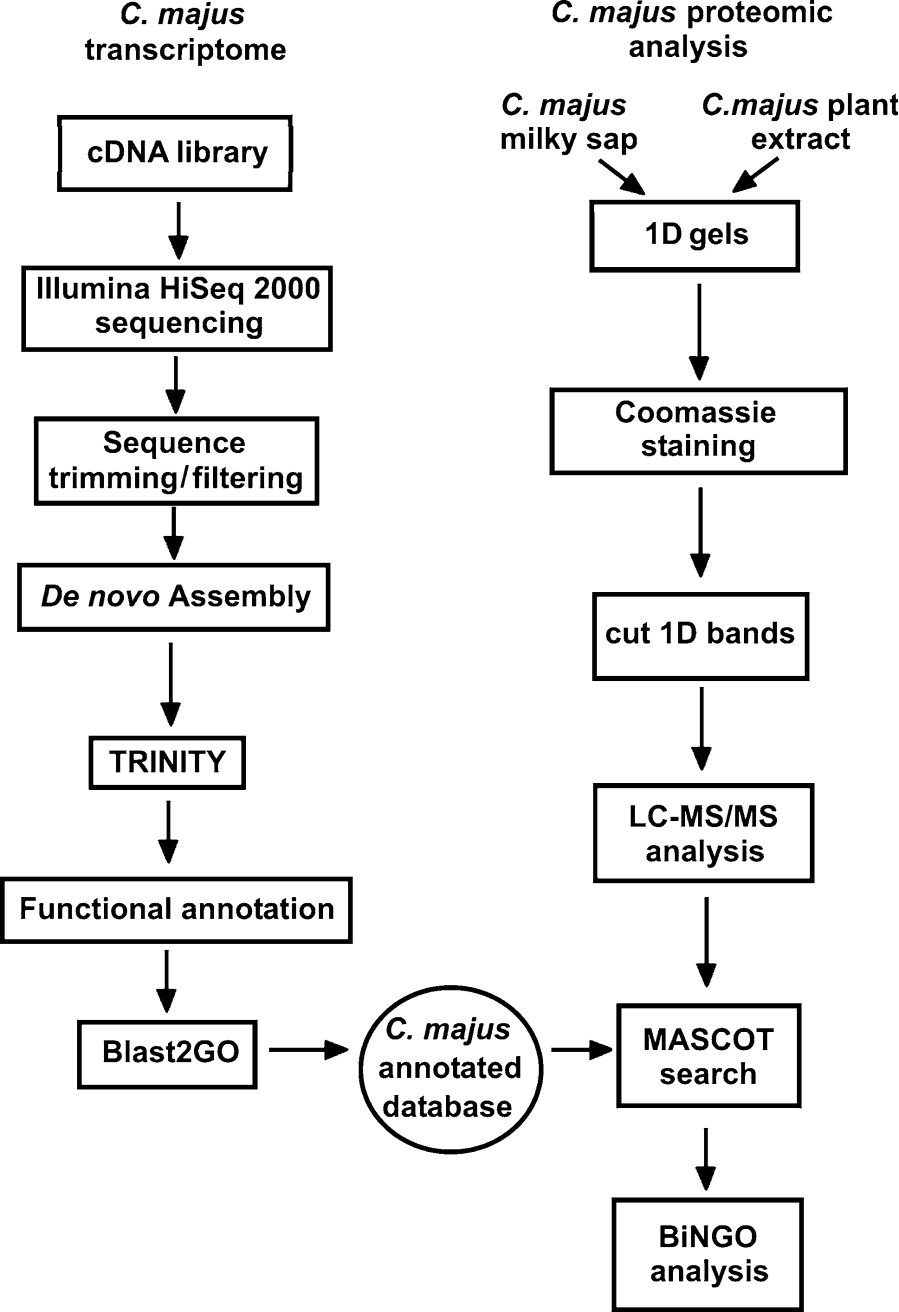
Schematic representation of the overall sequencing and annotation workflow for *C. majus* transcriptome together with proteomic analysis

The aim of the present study was to sequence, assemble, and annotate the *C. majus* transcriptome. We created a database for protein identification for mass spectrometry data. The database allowed us to significantly improve the sensitivity of previous assessments concerning composition of *C. majus* milky sap, which relied only on NCBInr (National Center for Biotechnology Information non-redundant) database searches with *Viridiplantae* filter (Nawrot et al. 2007a, b, 2014). A schematic representation of the overall sequencing, annotation, and protein identification workflow is presented in Fig. 2. The integration of transcriptomic and proteomic data in this study shows that it is possible to answer the emerging biological issues on non-model plant species without genome sequence available, such as novel insights into plant latex protein composition.

## Materials and methods

### Plant material

*Chelidonium majus* L. plants were planted in IPK Gatersleben green house from *C. majus* seeds collected in Poznań, Poland, on 12 June 2012. The voucher specimen of seeds is deposited in the Department of Molecular Virology, Faculty of Biology, Adam Mickiewicz University in Poznań, Poland. They were cultivated in small cuvette with pH 5.5 ground, and after 4 weeks, they were transferred to bigger pots. Supplemented with mineral substrates (N 6 g/m^2^, P_2_O_5_ 7 g/m^2^, K_2_O 9 g/m^2^), they were cultivated until reaching the height of ca. 25–30 cm for 6 weeks.

### Illumina HiSeq 2000 sequencing

*Chelidonium majus* RNA samples were isolated from stem of the plant with exuding milky sap (Fig. 1) using RNeasy Plant Mini Kit (Qiagen). cDNA synthesis was carried out and cDNA library was prepared using TruSeq RNA Library Prep Kit v2 (Illumina Inc., San Diego, CA, USA). After RNA quality validation on Agilent Technologies 2100 Bioanalyzer, the library was sequenced using Illumina HiSeqTM 2000 (Illumina Inc.).

### *De novo* transcriptome assembly

Paired RNA-seq reads were pre-trimmed at the first undetermined base or at the first base having Phred quality below 20. The pairs with one (or both) reads shorter than 31 bases after trimming were excluded from the assembly process. In total, 112,544,804 (84 %) out of 133,550,365 read pairs passed filtering. Subsequently, the transcripts were assembled *de novo* using Trinity r2013-02-25 (specifically designed to deal with *de novo* transcriptome assembly, with default settings) (Grabherr et al. 2011). Protein coding sequences (CDS) were predicted using TransDecoder (http://transdecoder.sourceforge.net/) from Trinity package. Non-redundant CDS were selected for annotation and further analyses. *De novo* assembly was performed by VitaInSilica (http://www.vitainsilica.pl/).

### Gene annotation and analysis

To obtain comprehensive information on the function of predicted CDS, we used Blast2GO that annotates sequences based on ontological data retrieved for top BLAST (Basic Local Alignment Search Tool) hits of each sequence. To generate the comparison, local BLASTx search with the e value cutoff of 1e-10 against complete RefSeq (NCBI Reference Sequence) protein database (release 64, available at NCBI FTP server from 14 March 2014) was performed. The resulting XML document was imported to Blast2GO software and refined by imposing more stringent cutoff (1e-25). After InterProScan analysis, the program assigned provisional names and gene ontology (GO) terms to annotated CDS. The additional BLAST analysis performed during annotation step allowed us to scrutinize sequences for remaining contaminants of non-plant origin. Additionally, KAAS [Kyoto Encyclopedia of Genes and Genomes (KEGG) Automatic Annotation Server] was used to functionally annotate genes in a genome by amino acid sequence comparisons against a manually curated set of ortholog groups from dicot plants in KEGG GENES (http://www.genome.jp/kegg/genes.html).

### One-dimensional gel electrophoresis (1-D, SDS-PAGE)

To verify the protein composition of protein samples, sodium dodecyl sulfate–polyacrylamide gel electrophoresis (SDS-PAGE) was carried out in a slab mini-gel apparatus according to Laemmli (1970), using 10 % polyacrylamide as the separating gel and 5 % polyacrylamide as the stacking gel. The proteins were reduced by heating them to 100 °C in the presence of 2-mercaptoethanol for 5 min. About 50 μg of each sample was added to the gel, including two technical replicates. After SDS-PAGE, the gels were fixed and stained using sensitive Coomassie blue staining (Neuhoff et al. 1988).

### Liquid chromatography and tandem mass spectrometry analysis (LC–MS/MS)

Gel bands were digested with trypsin and, finally, all were subjected to LC–ESI-MS/MS analysis in Mass Spectrometry Laboratory, Institute of Biochemistry and Biophysics, Polish Academy of Sciences, Warsaw, Poland. Tryptic peptide mixtures were analyzed by LC–MS/MS using nanoflow HPLC and a Linear Trap Quadrupole (LTQ) Orbitrap XL mass spectrometer (Thermo Fisher Scientific) as the mass analyzer. Peptides were eluted from a 75-µm analytical column on a linear gradient from 10 to 30 % acetonitrile over 50 min and sprayed directly into the LTQ-Orbitrap mass spectrometer.

### Tandem mass spectrometry (MS/MS) data analysis

Proteins were identified by MS/MS via information-dependent acquisition of fragmentation spectra (Nawrot et al. 2007a, 2014) using MASCOT 2.4.1 search (Perkins et al. 1999; Matrix Science, London, UK; www.matrixscience.com) against *C. majus* CDS database.

### Quantitative comparative analysis of *C. majus* milky sap proteins

For quantitative, comparative analysis of *C. majus* milky sap with *C. majus* whole plant samples based on emPAI (exponentially modified protein abundance index) values of individual proteins, we analyzed data from three independent specimens of latex and three corresponding whole plant extracts. emPAI value is proportional to protein abundance in a protein mixture (Ishihama et al. 2005). Approximate relative abundance of each protein (calculated from emPAI according to formula: (emPAI value × 100)/sum of all emPAI values in the category) was averaged among three latex replicates and compared with the corresponding value calculated for whole plant extract. The results with a sap/extract ratio >2 and *t* test significance level <0.1 were considered as significantly overrepresented in the sap.

### Biological networks gene ontology tool (BiNGO) analysis

The overrepresentation of ontological terms in tested set (milky sap) compared to whole plant extract proteome and transcriptome was assessed using BiNGO tool. BiNGO (Maere et al. 2005) is a Cytoscape (Saito et al. 2012) plugin determining which GO categories are statistically over- or underrepresented in a set of genes. It maps the predominant functional themes of a given gene set on the GO hierarchy and outputs this mapping as a Cytoscape graph.

## Results

### Illumina sequencing and *de novoC. majus* transcriptome assembly

**Fig. 3:**
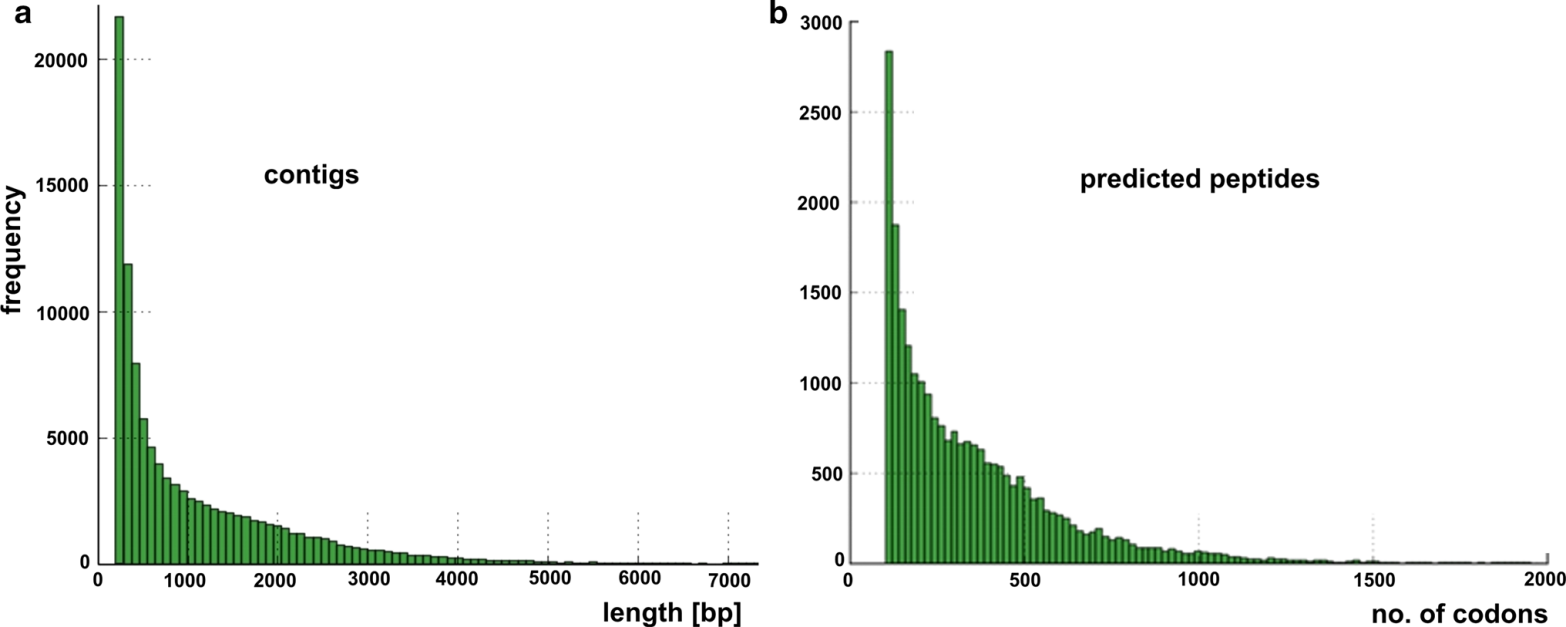
*Chelidonium majus* transcriptome assembly overview. **a** Length distributions of assembled contigs. **b** Length distributions of predicted peptides (peptide lengths for raw transcripts): mean 356.44, standard deviation 267.36. Data obtained after Illumina paired-end sequencing of the normalized 6-week-old stem cDNA library

With the purpose of understanding *C. majus* transcriptome, RNA was extracted from the young stem of 6-week-old *C. majus* plant with exuding milky sap (Fig. 1c) and sequenced with Illumina paired-end sequencing technology generating approximately 119 Mb of raw sequence data from 133,550,365 reads with an average length of almost 100 bp (Fig. 3; Table 1).

**Table 1:** Summary of *C. majus* transcriptome assembly and annotation

Assembly		Annotation	
Contigs	107,088	CDS	52,896
Cumulative assembly size	119,198,595	Complete	35,271
GC (%)	39.27	3′-partial	4182
Contigs >1 kb	41,454	5′-partial	9914
Bases in contigs >1 kb	90,034,432	Internal	3529
N50	1913	Unique	34,965
N90	450	Contaminant	33
Number of gaps	0	Remaining	34,932
Longest	15,519	Annotated	23,004

N50 and N90 define the length of contig for which the set of contigs with greater or equal length corresponds to 50 and 90 % of the assembly, respectively. CDS were classified as complete (both start and stop codon present), 3′-partial (only stop codon), 5′-partial (only start codon), and internal (neither start nor stop codon present) CDS. Unique CDS are all non-redundant CDS (regardless of completeness) detected during the study

After the quality and adaptor trimming process, *de novo* transcriptome assembly was performed using Trinity suit (specifically designed to deal with *de novo* transcriptome assembly) (Grabherr et al. 2011). This resulted in 107,088 contigs, with N50 of 1913 bp and N90 of 450 bp (Table 1).

### Annotation of *C. majus* transcriptome

TransDecoder detected 52,896 CDS including 35,271 (66.68 %) complete ones (Table 1). In addition, 9137 RNAs with length over 1 kb with no coding potential (lncRNAs) were assembled suggesting significant presence.

**Fig. 4:**
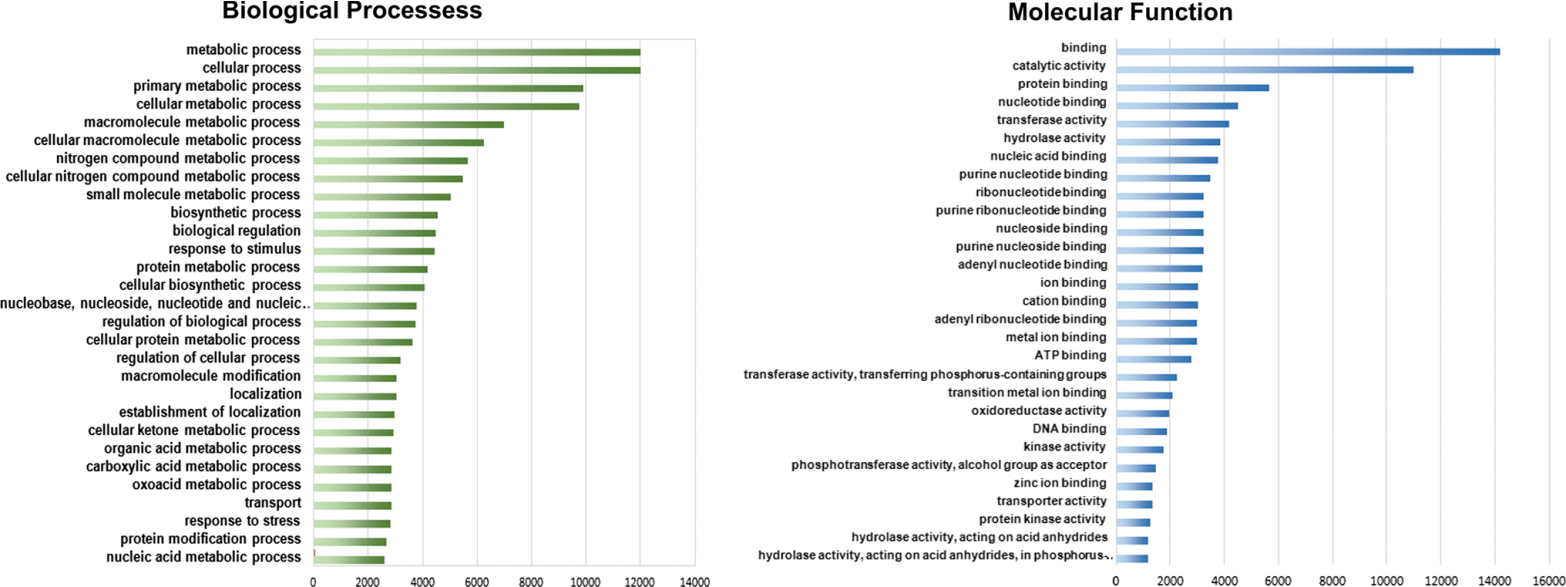
GO distributions for *C. majus* transcriptome. Main functional categories in the biological process (BP, level four) and molecular function (MF, level three) found in the transcriptome relevant to plant physiology

Detailed analysis of BLAST results revealed that 33 of coding sequences (0.09 %) may be contaminants of either fungal or bacterial origin (likely transcripts of plant commensal flora). These sequences were removed from the analyzed set. About 89.07 % of the remaining unique CDS had at least one BLAST hit against RefSeq database if e value cutoff equal to 1e-10 was applied. Proportion dropped to 82.09 % when more stringent cutoff (1e-25) was imposed. InterProScan utility found protein domains in 89.32 % CDS. About 35.98 % (12,692) sequences were annotated using KAAS with KO (KEGG Orthology) assignments and automatically generated KEGG pathways (Supplementary Table S1). About 65.85 % (23,004) sequences were annotated by Blast2GO with provisional names and GO categories (Table 1, Supplementary Table S1). Among them, 57.00 % was associated with molecular function, 46.42 % with biological processes, and 37.26 % was assigned to cellular components (Fig. 4; Supplementary Table S2). GO terms associated with primary metabolites were found, such as universal building blocks of sugars, amino acids, nucleotides, lipids, and energy sources. In addition, GO terms associated with macromolecule metabolic processes (6995), small molecule metabolic processes (5042), and protein metabolic processes (4189) were found. Analyzing the obtained transcripts, we found sequences connected to major branches of metabolism and signal transduction that are expected in complex systems (Garzon-Martinez et al. 2012). Brief summary of GO annotation is shown in Fig. 4. It is noteworthy that we found 4424 transcripts that fall into “response to stimulus” category (BP level 2), as this group includes candidate genes for pathogen resistance and many putative components of the milky sap. All unique coding sequences were used to construct reference database for further mass spectrometry analysis.

### Analysis of overrepresented GO terms in *C. majus* latex

**Fig. 5:**
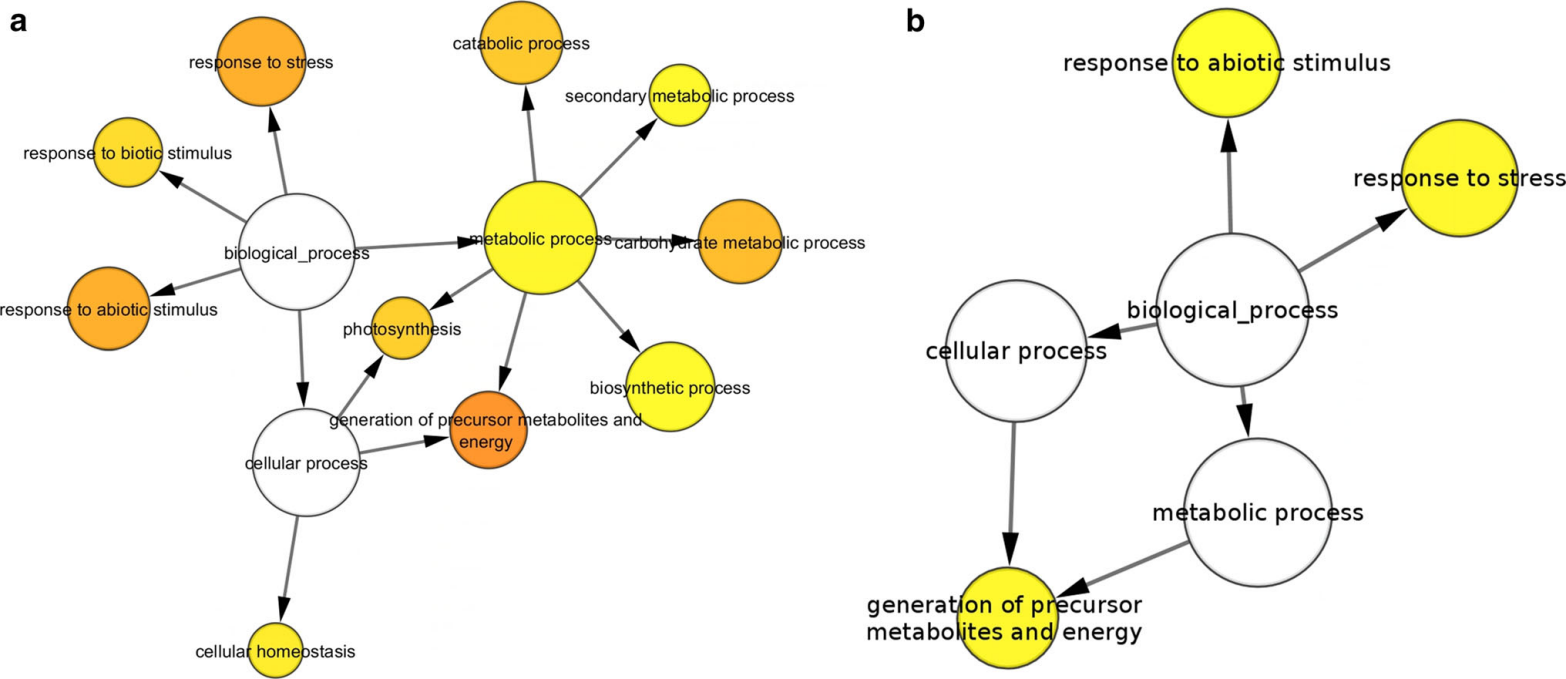
Results of comparison of *C. majus* proteomic samples using BiNGO. **a** Milky sap proteome versus transcriptome (CDS database) (significance level 0.005). **b** Milky sap proteome versus whole plant extract proteome (significance level 0.05)

We utilized BiNGO to compare GO terms between *C. majus* milky sap and *C. majus* whole plant extract proteomes. The analysis comprised two steps. The first step was the comparison of *C. majus* latex proteome to *C. majus* transcriptome (Fig. 5a). The abundance of proteins involved in response to stress, response to biotic stimulus, response to abiotic stimulus, secondary metabolic process, and cellular homeostasis remains in agreement with the putative protective function of the milky sap. On the contrary, it is much harder to explain overrepresentation of photosynthesis, carbohydrate metabolic process, or generation of precursor metabolites and energy.

Second step was the comparison of latex against whole plant extract proteomes. The results (Fig. 5b) confirmed overrepresentation of response to biotic stimulus, response to stress, and generation of precursor metabolites and energy terms.

### Analysis of *C. majus* protein composition

Previous functional assignments of *C. majus* proteins relied on homology-based information, because the sequence information of this plant was not available (Remmerie et al. 2011; Nawrot et al. 2007a, b, 2014). The novel transcriptome-based *C. majus* database allowed us to significantly improve the sensitivity of previous proteomic assessments concerning composition of *C. majus* milky sap and extracts and provide meaningful biological-based information.

For proteomic assessments using the novel database, we used two types of datasets. One of them was previously published data for proteomic content of *C. majus* whole plant extracts using an approach of protein separation by 1-D SDS-PAGE to subsequent LC–MS/MS analysis of individual gel bands (i.e., shotgun approach) (Nawrot et al. 2014). The main advantages of such approach are sample purity after SDS-PAGE improving proteome coverage of analyzed samples (Schulze and Usadel 2010; Matros et al. 2011). The other dataset comprised *C. majus* milky sap samples analyzed using the same approach. For this purpose, we prepared three independent specimens of latex (isolated from stems of 6- to 8-week-old plants) and applied shotgun approach to identify proteins (1-D gel electrophoresis, subsequent whole lane trypsin digestion and LC–ESI-MS/MS analysis). For both types of datasets, raw proteomic data were searched against annotated *C. majus* CDS database using Mascot software. In total, 334 different putative proteins were identified for *C. majus* milky sap (for all three replicates) and 1155 for *C. majus* whole plant extract (Supplementary Tables S3, S4). The complexity of proteins in *C. majus* milky sap is hence about 3.5 times lower than in whole plant extract. The number of identified proteins from both milky sap and whole plant extract and unique CDS from transcripts were assigned to most represented KEGG pathways in *C. majus* and compared in Supplementary Table S5.

## Discussion

### *Chelidonium majus* milky sap protein composition based on comparative analysis

To demonstrate the power of the use of our new *C. majus* CDS database, we performed quantitative, comparative analysis of *C. majus* milky sap and whole plant protein samples based on emPAI obtained during Mascot search (Supplementary Table S3) to show proteins which are overrepresented in the latex, hence potentially “sap-specific”. The average of relative abundance of each protein of three latex replicates was compared with corresponding value calculated for three whole plant extract replicates (Supplementary Table S4). The resulting ratio informs about the level of overrepresentation of identified protein in the specified type of sample. The proteins observed in all sap samples with a sap/extract ratio >2 and *t* test significance level <0.1 were considered as significantly overrepresented in *C. majus* milky sap. Overall results of the analysis are present in Supplementary Table S4. Approximately, 29 of 334 initially identified proteins met the above-mentioned criteria and were classified as overrepresented in the milky sap (Table 2).

**Table 2:** Proteins overrepresented in *C. majus* milky sap comparing to whole plant extract

No.	Transcript name	Protein name	Mean % emPAI milky sap	Mean % emPAI whole plant extract	Mean milky sap/mean whole plant extract ratio	*T* test, *P* values* <0.1
Stress and defense response						
1	m.37895	Mlp-like protein 28	10.32	0.00	Only in the sap	0.092
2	m.37899	Mlp-like protein 28	9.93	0.00	Only in the sap	0.047
3	m.37901	Mlp-like protein 28	2.66	0.00	Only in the sap	0.093
4	m.12634	Mlp-like protein 28	2.39	0.00	Only in the sap	0.093
5	m.12629	Mlp-like protein 34	1.73	0.03	49.91	0.094
6	m.60893	Polyphenol oxidase	1.21	0.04	32.76	0.080
7	m.12632	Mlp-like protein 28	1.05	0.01	73.34	0.094
8	m.2644	Polyphenol oxidase	0.83	0.00	Only in the sap	0.008
9	m.62963	Polyphenol chloroplastic-like	0.18	0.00	Only in the sap	0.093
10	m.18123	Mlp-like protein 28	0.12	0.00	Only in the sap	0.092
Generation of precursor metabolites and energy						
11	m.7838	Reticuline oxidase-like	3.73	0.05	78.19	0.073
12	m.28580	Probable caffeoyl-*o*-methyltransferase at4g26220-like	1.90	0.00	Only in the sap	0.071
13	m.2541	Anthocyanidin-o-glucosyltransferase-like	1.01	0.00	Only in the sap	0.061
14	m.44303	Reticuline oxidase-like	0.68	0.00	Only in the sap	0.062
15	m.44308	Reticuline oxidase-like	0.26	0.00	Only in the sap	0.099
16	m.61702	Protein usf-like	0.15	0.00	Only in the sap	0.098
Stress and defense response—redox, antioxidant defense system						
17	m.60799	Lactoylglutathione lyase-like	1.45	0.16	9.13	0.013
18	m.7868	Peroxidase 12-like	2.83	0.00	Only in the sap	0.061
19	m.14064	Protein in 2-1 homolog b-like	2.15	0.04	56.39	0.057
20	m.2578	Peroxidase 12-like	0.55	0.05	10.77	0.043
21	m.63454	Quinone oxidoreductase 1-like	0.51	0.05	10.95	0.079
22	uniq_03745	Isoflavone reductase homolog	0.15	0.00	Only in the sap	0.098
Storage and others						
23	m.54853	Beta-amylase precursor	5.83	0.17	34.17	0.038
24	m.18198	Ubiquitin-40s ribosomal protein s27a-like	3.57	0.29	12.52	0.037
25	m.60713	Lipoxygenase homology domain-containing protein 1-like	1.48	0.10	14.31	0.007
26	m.60734	Lipoxygenase homology domain-containing protein 1-like	1.10	0.11	9.64	0.030
Not assigned						
27	m.61326	UNCHARACTERIZED_PROTEIN	2.49	0.22	11.45	0.040
28	m.2643	UNCHARACTERIZED_PROTEIN	0.87	0.00	Only in the sap	0.092
29	m.60859	UNCHARACTERIZED_PROTEIN	0.81	0.06	12.98	0.050

Protein identification results were presented according to their relative abundance in the latex comparing to relative abundance of the same protein hits in *C. majus* whole plant extract (ratio of the average of the relative emPAI value of the latex and whole plant extract). Proteins were selected according to *t* test values (*P* < 0.1), the presence of identified proteins in at least two of three sap replicates, mean sap/extract ratio >2, and mean % emPAI milky sap values >0.1. The list of all overrepresented proteins is enclosed in Supplementary Table S4

* *t* test was used to determine if the emPAI values of selected identified proteins are significantly different between *C. majus* milky sap (*n* = 3) and *C. majus* whole plant extract samples (*n* = 3) with *P* < 0.1

### Stress- and defense-related proteins overrepresented in *C. majus* milky sap

One of the overrepresented, highly abundant proteins identified only in *C. majus* milky sap, but not in *C. majus* whole plant extract, was MLP-like protein 28, which belongs to major latex protein (MLP) class (Table 2, nos. 1–4, 7, 10). It accounted for more than a quarter (26.47 %) of the protein content of the sap belonging to stress- and defense-related proteins. The second major latex protein identified was MLP-like protein 34 (Table 2, no. 5), which accounted for 1.73 % of the protein content of the milky sap. The major latex protein/ripening-related protein (MLP/RRP) subfamily is the second largest subfamily among plant proteins with 60 members, 31 of them are from *Arabidopsis thaliana* (L.) Heynh. (Radauer et al. 2008). The members of this subfamily were first described as proteins abundantly expressed in the latex of opium poppy (*Papaver somniferum* L.) (Nessler and Burnett 1992; Decker et al. 2000). The biological function of the MLP/RRP proteins is still unknown, but they have been associated with fruit and flower development and in defense or stress response (Radauer et al. 2008). Based on the modest sequence similarity, they have been characterized as members of the Bet v1 protein superfamily (Bet_v_I [Pfam:PF00407]) (Lytle et al. 2009). The most distinctive feature of the Bet v1 fold is a large solvent accessible hydrophobic cavity, which may function as a ligand-binding site (Radauer et al. 2008).

The other stress- and defense-related protein found in *C. majus* milky sap, which accounts for 2.22 % of the protein content of the sap, is polyphenol oxidase (PPO), which is present mainly in the latex (Table 2, nos. 6, 8–9). Two unigenes of PPO were found in *C. majus* latex: one of them is present only in the sap (Table 2, no. 8) and second is ca. 33 times more abundant in the sap than in whole plant extract (32.76-fold, Table 2, no. 6). Polyphenol oxidases or tyrosinases (PPO), also known and reported under various names (phenolase, catechol oxidase, catecholase, monophenol oxidase, *o*-diphenol oxidase, and orthophenolase) based on substrate specificity, are widely distributed in plants and fungi (Wititsuwannakul et al. 2002). The activation of PPO leads to the oxidation of phenolic compounds, consequently enhancing the resistance. PPO is also involved in the generation of reactive oxygen species (ROS). It was observed that exuding yellow *C. majus* milky sap becomes brown after exposure to air. Such browning reactions are caused by the presence of PPO family genes (Mayer 2006). Other data show that latex of different plants coagulates when exposed to air. Therefore, it is proposed that natural latex has a protective function, sealing wounds, acting as a barrier to microorganisms, and discouraging herbivory (El Moussaoui et al. 2001; Wahler et al. 2009). The consistency of latex itself has a defensive role because the glue-like exudate seals wounds in the plant from pathogen attack and coats the mouthparts of herbivores (Hagel et al. 2008; Konno 2011; Escalante-Perez et al. 2012).

### Alkaloid and secondary metabolite biosynthetic proteins in the milky sap

*Chelidonium majus* latex is rich in a range of secondary metabolites such as alkaloids, flavonoids, or phenolic acids. *C. majus* contains alkaloids such as chelidonine, sanguinarine, cheleritrine, and berberine (Colombo and Bosisio 1996). Therefore, we identified enzymes involved in biosynthesis of these compounds: anthocyanidin-*o*-glucosyltransferase-like (Table 2, no. 13), probable caffeoyl-o-methyltransferase At4g26220-like (Table 2, no. 12) and reticuline oxidase-like (Table 2, nos. 11, 14–15).

Anthocyanidin-*o*-glucosyltransferase-like is an enzyme involved in anthocyanidin biosynthesis, which belongs to flavonoids. Anthocyanidins are common plant pigments. They are the sugar-free counterparts of anthocyanins forming a large group of polymethine dye (Mouradov and Spangenberg 2014).

Caffeoyl-CoA *O*-methyltransferase (EC 2.1.1.104) is an enzyme that catalyzes the reaction of conversion of *S*-adenosyl-l-methionine with caffeoyl-CoA into *S*-adenosyl-l-homocysteine and feruloyl-CoA. A large number of natural products are generated via a step involving this enzyme, which participates in phenylpropanoid biosynthesis (Boerjan et al. 2003).

Reticuline oxidase-like is the berberine bridge enzyme [(*S*)-reticuline: oxygen oxidoreductase (methylene-bridge-forming), EC 1.5.3.9], a vesicular plant enzyme that catalyzes the reaction along the biosynthetic pathway that leads to benzophenanthridine alkaloid biosynthesis. (*S*)-Reticuline is a key branch-point intermediate that can be directed into several alkaloid subtypes with different structural skeleton configurations (Ziegler et al. 2009). Cytotoxic benzophenanthridine alkaloids are accumulated in certain species of Papaveraceae and Fumariaceae in response to pathogenic attack and, therefore, function as phytoalexins (Dittrich and Kutchan 1991).

### Antioxidant and metabolic proteins in *C. majus* latex

The analysis confirmed the presence of the protein components of antioxidant defense system in the *C. majus* latex. These proteins form the first line of defense against different stress conditions and help to prevent from attack of different pathogens (Walz et al. 2002), which are highly abundant in the milky sap (Table 2, nos. 17–22). Peroxidase 12-like (Table 2, no. 18) and isoflavone reductase homolog (Table 2, no. 22) were present only in the milky sap. The presence of class III plant peroxidase, glyoxalase, quinone reductase, and ubiquitin in *C. majus* latex was previously reported (Nawrot et al. 2007a, b).

*Chelidonium majus* latex also contains metabolic and storage proteins (Table 2, nos. 23–26). Beta-amylase precursor was relatively abundant (no. 23; 5.83 %), with 34.17-fold overrepresentation in the milky sap comparing to whole plant extract. Beta-amylase is an enzyme which hydrolyzes glucans derived from starch granules to maltose and is present in different plant organs (Fulton et al. 2008). Its presence in the milky sap could be explained by the differentiation of articulated laticifers of *C. majus*. Such gradual degeneration of the cytoplasm occurs in many species and is often accompanied by the appearance of altered plastids and characteristic starch grains (Hagel et al. 2008).

## Conclusions

Our study presents *de novo* assembly and characterization of *C. majus* transcriptome. Further comparative proteomic analysis of *C. majus* milky sap and whole plant extract samples provided new insights into milky sap protein composition. The novel transcriptome-based *C. majus* database allowed to significantly improve the sensitivity of proteomic identifications. In the present study, 334 different putative proteins were identified in *C. majus* milky sap samples comparing to only 21 in previous study (Nawrot et al. 2007a, b). Moreover, our approach enabled identification of the major latex protein (MLP) 28, which could not be detected without species-specific database. The quantitative analysis confirmed that *C. majus* latex is rich in proteins connected with response to stress conditions and generation of precursor metabolites and energy. We also identified polyphenol oxidase (PPO), several enzymes involved in biosynthesis of natural products and a range of abundant antioxidant proteins. These findings support the importance of *C. majus* latex for plant defense against pathogens and herbivores.

The obtained *C. majus* annotated CDS database will serve as a valuable dataset for further studies of *C. majus* proteomes and as comparative material for other plant species.

The data sets supporting the results of this article are available at the NCBI Sequence Read Archive (SRA) and are available under SRA Accession Number SRR1998045 (related BioProject PRJNA264791—*Chelidonium majus* transcriptome, related BioSample SAMN03142649).

### *Author contribution statement*

RN performed the study, collected plant material, performed proteomic analyses and prepared the manuscript. JB performed gene annotation, bioinformatics and comparative analyses. RL participated in design and coordination of the study, collected plant material. LA performed sequencing and initial assembly. HPM supervised the work, participated in its design and coordination and corrected the manuscript. All authors read and approved the final manuscript.

## Electronic supplementary material

Below is the link to the electronic supplementary material.

Supplementary material 1 (XLSX 10396 kb)

Supplementary material 2 (XLSX 16 kb)

Supplementary material 3 (XLSX 326 kb)

Supplementary material 4 (XLSX 331 kb)

Supplementary material 5 (XLSX 21 kb)

## Abbreviations

CDS: Coding sequences
GO: Gene ontology
